# Successful Treatment of Homozygous Familial Hypercholesterolemia Using Cascade Filtration Plasmapheresis

**DOI:** 10.5152/tjh.2011.20

**Published:** 2011-04-01

**Authors:** Fatih Kardaş1, Aysun Çetin, Musa Solmaz, Rüksan Büyükoğlan, Leylagül Kaynar, Mustafa Kendirci, Bülent Eser, Ali Ünal

**Affiliations:** 1 Erciyes University, School of Medicine, Department of Pediatric Metabolism, Kayseri, Turkey; 2 Erciyes University, School of Medicine, Department of Biochemistry, Kayseri, Turkey; 3 Erciyes University, School of Medicine, Department of Hematology, Kayseri, Turkey

**Keywords:** Familial hypercholesterolemia, Cascade filtration plasmapheresis, Low-density lipoprotein cholesterol (LDL-C)

## Abstract

**Objective:** The aim of this study was to report the efficacy of low-density lipoprotein cholesterol (LDL-C) apheresisusing a cascade filtration system in pediatric patients with homozygous familial hypercholesterolemia (FH), and toclarify the associated adverse effects and difficulties.

**Material and Methods:** LDL-C apheresis using a cascade filtration system was performed in 3 pediatric patientswith homozygous FH; in total, 120 apheresis sessions were performed.

**Results:** Cascade filtration therapy significantly reduced the mean LDL-C values from 418 ± 62 mg/dL to 145 ± 43 mg/dL (p= 0.011). We observed an acute mean reduction in the plasma level of total cholesterol (57.9%), LDL-C (70.8%),and high-density lipoprotein cholesterol (HDL-C) (40.7%). Treatments were well tolerated. The most frequent clinicaladverse effects were hypotension in 3 sessions (2.5%), chills (1.7%) in 2 sessions, and nausea/vomiting in 3 sessions(2.5%).

**Conclusion:** Our experience using the cascade filtration system with 3 patients included good clinical outcomes andlaboratory findings, safe usage, and minor adverse effects and technical problems.

**Conflict of interest:**None declared.

## Abstract (tr)

**Amaç:** Çalışmamızın amacı, homozigot ailevi hiperkolesterolemili çocuk hastalarda ‘cascade filtration’ tekniği ile yapılan düşük dansiteli lipoprotein kolesterol (LDL-C) aferez tedavisinin etkinliğinin tespit edilmesi, işlem sırasında oluşabilecek yan etkilerin ve zorlukların ortaya konulmasıdır.

**Gereç ve Yöntemler:** Homozigot ailevi hiperkolesterolemili 3 çocuk hastada, ‘cascade filtration’ sistemi kullanılarak LDL aferezi uygulandı. Tüm hastalar için toplam 120 aferez seansı yapıldı.

**Bulgular:** ‘Cascade filtration’ tedavisi ile ortalama LDL-C seviyeleri, 418 ± 62 mg/dl’den 145 ± 43 mg/dl’ye belirgin olarak düştü (p=0.011). Total kolesterol, LDL-kolesterol ve HDL-kolesterol’ün plazma seviyelerindeki akut ortalama düşüş sırasıyla, %57.9, %70.8, %40.7 olarak bulundu. Tedaviler iyi tolere edildi. Klinik olarak en sık görülen yan etkiler, 3 seansta hipotansiyon (%2,5), 2 seansta titreme/üşüme (%1,7) ve 3 seansta bulantı/kusma (%2,5) idi.

**Sonuç:** ‘Cascade filtration’ sistemi ile 3 hastamızın tedavileri sonucunda, yüz güldürücü klinik bulgular ve laboratuvar sonuçları, güvenli kullanım, yok denecek kadar az yan etki ve teknik problemler tespit edilmiştir.

## INTRODUCTION

Familial hypercholesterolemia (FH) is an autosomaldominant inherited disorder characterized by elevatedserum low-density lipoprotein cholesterol (LDL-C). Patientswith FH exhibit 2 distinct syndromes, depending onwhether the LDL receptor gene is present in the heterozygousor homozygous form. The frequency of the heterozygousform is 1:500 and that of the homozygous form is1:1,00,000 [1]. The most frequent genetic defect is a mutationon the LDL receptor gene, and other rare mutationsoccur on the ApoB and PCSK9 genes [2]. Clinical manifestationsdevelop earlier in the homozygous form andinclude tendon xanthomata, arcus cornea, and prematureheart disease [1]. Homozygotes develop planar xanthomason the fingers and toes by the age of 5 years [3]. Coronaryheart disease (CHD), and aortic root stenosis develop duringadolescence, and myocardial infarction (MI) developduring the third decade. Heterozygotes develop xanthomasduring the fourth decade.

Heterozygous patients can be treated effectively withdiet and drugs; however, homozygous patients do not respondto these therapeutic interventions [4,5]. In patientswith homozygous FH, complications cannot be delayedusing these interventions and when they are unsuccessfulselective LDL-C apheresis should be considered [6,7]. Ithas been proven that long-term reduction in risk factorscan result in regression of coronary stenosis, and in reducedcoronary morbidity and mortality [8]. Recent studiesindicate that weekly or bi-weekly plasmapheresis—preferably LDL apheresis—is the cornerstone of treatmentin the majority of children with homozygous FH [9].

Several plasma-apheresis methods have been used inthe treatment of homozygous FH, such as Liposorber D,direct adsorption of lipoproteins (DALI), and cascade filtration[10,11,12]. Membrane differential filtration (MDF)(cascade filtration) is another apheresis technique withwhich atherogenic lipoproteins can be effectively eliminatedfrom plasma [4,5]. With MDF apheresis LDL particles, lipoprotein(a) [LP(a)], and fibrinogen, which exhibit alarge molecular diameter and a high molecular weight, arepreferentially retained in the filter and are thus eliminatedfrom the plasma; however, high-density lipoprotein (HDL)particles and other high molecular weight proteins (e.g.immunoglobulins) are also retained, to a certain degree.MDF apheresis, therefore, is a semi-selective LDL apheresistechnique [13]. Herein, we present 3 homozygousFH patients treated with LDL apheresis (cascade filtrationtechnique), and a discussion in light of the literature.

## MATERIALS AND METHODS

**Patients**

**Patient 1 **

A 13-year-old boy admitted to our clinic because his mother was concerned about brown skin lesions on his elbows, knees, and Achilles tendons (Figure 1a). The primary xanthomas first appeared on his knees and elbows when he was 5 years old. His parents were not consanguineous. His father was diagnosed with FH and died of MI due to CHD at 25 years of age. His mother’s blood lipid levels were in the normal range. The boy was diagnosed as FH at another clinic 1 year before presentation and was intermittently treated with atorvastatin 20 mg d–1 for 1 year, but an effective reduction in LDL-C was not achieved. 

The patient’s growth and developmental history was normal. Physical examination showed multiple brown xanthomas on his elbows, knees, and Achilles tendons. The results of cardiovascular examinations, including echocardiography, were normal. Laboratory investigation results were as follows: total cholesterol: 895 mg/dL; LDLC: 828 mg/dL; HDL cholesterol (HDL-C): 39 mg; triglycerides: 140 mg/dL; fibrinogen: 324 mg/dL; immunoglobulin (Ig)G: 702 ng dL–1; IgM: 78 mg/dL; IgA: 118 mg/dL. He was started on a diet low in saturated fat and cholesterol, and a cholesterol-lowering drug combined with cascade filtration apheresis therapy. 

**Patient 2**

A 15-year-old male patient admitted to our clinic due to yellow skin lesions. The lesions first appeared on his knees when he was 8 years old. He had not been examined or treated prior to presentation. Physical examination showed multiple xanthomas on his knees, elbows, and sacral region. Cardiovascular system examination and echocardiography were normal. Laboratory examination results were as follows: total cholesterol: 731 mg/dL; LDLC: 660 mg/dL; HDL-C: 33 mg/dL; triglycerides: 188 mg/ dL; fibrinogen: 281 mg/dL; IgG: 803 mg/dL; IgM: 129 mg/ dL; IgA: 124 mg/dL. The patient was clinically diagnosed as homozygous FH. He was started on a diet similar to the previous patient and simvastatin 20mg d–1, and was followed-up. After 6 months of treatment no regression in blood lipid levels was observed and the clinical features persisted. Apheresis therapy was then added to the therapeutic regimen. 

**Patient 3**

An 18-year-old male patient (brother of patient 2) presented to our clinic with the same complaints as patient 2. Physical examination showed multiple xanthomas on his knees, elbows, sacral region, and Achilles tendons; the remainder of the examination was normal. Laboratory test results were as follows: total cholesterol: 767 mg/dL; LDLC: 706 mg/dL; HDL-C: 35 mg/dL; fibrinogen: 303 mg/dL; IgG: 787 mg/dL; IgM: 136 mg/dL; IgA: 128 mg/dL. He was clinically diagnosed as homozygous FH. The same conventional treatment given to patient 2 was started and he was followed-up for 6 months. The therapeutic regimen, including diet and cholesterol-lowering therapy, was not effective and LDL apheresis treatment was added to the treatment regimen. 

The parents of patients 2 and 3 were consanguineous. The mother’s laboratory investigation results were as follows: total cholesterol: 278 mg/dL; LDL-C: 207 mg/dL; HDL-C: 43 mg/dL; triglycerides: 140 mg/dL. The father’s results were as follows: total cholesterol: 331 mg/dL; LDLC: 262 mg/dL; HDL-C: 38 mg/dL; triglycerides: 165 mg/ dL. The parents’ laboratory results showed heterozygous FH. The patients’ clinical and biological data at presentation are summarized in Table 1.

**Methods**

**LDL-C apheresis **

**Figure 2:**
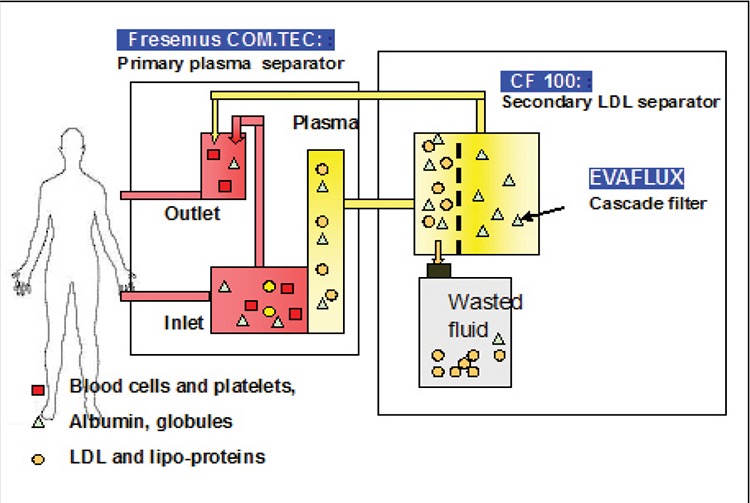
Schematic appearance of cascade filtration system,consisting of two separators.

All 3 patients received bi-weekly cascade LDL-C apheresis treatment at the Apheresis Unit of the Hematology Department. The semi-selective apheresis process, developed by Dr. Branger, consists of 2 separation systems. The primary separator device (Fresenius HemoCare, ComTec, Germany) separates plasma from whole blood. A secondary separator device (FC100, Infomed, Geneva, Switzerland) monitors the cascade filter by circulating plasma received from the primary separator and fractionates large proteins, such as LDL-C and fibrinogen (Figure 2). The primary plasma separation was performed using a plasma exchange kit (code: PL1, Fresenius). LDL-C was extracted from plasma product using a tubing set (TU-810-01, Infomed, Geneva, Switzerland) equipped with a fiber cascade filter (membrane surface: 2.0 m^2^; maximum pres sure: 66.6 kPa [500 mmHg]) (Evaflux model 5A20, code: 100205, Kawasumi Lab, Inc., Tokyo, Japan).

After attachment of the device via 2 venous access points in the antecubital veins, patient blood was pumped to the plasma filter with a continous flow rate. (30-50 mL min^—1^) to the plasma filter. The plasma product was then sent to the plasma bag of the adapter tubing set while controlling the plasma flow rate (15-30 mL min^–1^). Subsequently, the plasma was pumped by a secondary separation device to the hollow fiber cascade filter. High molecular weight proteins and lipoproteins were retained in its hollow fibers. Plasma and proteins with a diameter <30 nm passed through the filter and were returned to the replacement bag of the primary set; these were then sent to the patient after being combined with the previously separated whole blood. During apheresis the outlet of the hollow fibers of the cascade filter is closed, but can be opened if the pressure in the filter exceeds 400 mmHg, so as to release retinate into a container. After normal saline rinsing of the filter, which is performed automatically, the outlet must be closed again. In the present study the whole blood flow rate was maintained at 30-50 mL min^–1^ and the extracorporeal blood volume was calculated to be 340 mL (160 mL in the primary set, 40 mL in the adapter set, and 140 mL in the filter). Volume depletion was supported by physiological saline via the primary separator. During these processes the vital functions of all patients were closely monitored. 

Before treatment, the primary separator was primed with physiological saline mixed with 1/10 (vol/vol) of acid citrate dextrose (ACD-A) solution and 25 mL of 8.4% sodium bicarbonate solution L^–1^. The other system was primed with 3 L of normal saline only. In order to achieve the desired postapheresis LDL-C concentration, processed blood volume requires to be 1.5 fold the calculated plasma volume. During each procedure patient plasma volume was calculated according to the following formula: body weight (kg) x 80 x {1 − [hematocrit (%) x 0.91]} (e.g. for a body weight of 35 kg it should be 35 x 80 x [(1 − (0.37 x 0.91)] = 1857.24 mL) for the adsorption methods. 

Lipoprotein analyses, whole blood counts, biochemical parameters, coagulation, and immunoglobulin levels were measured immediately before and after each LDL apheresis session. All laboratory analyses were performed in the Core Laboratory of the Medical Faculty using routine measurements. 

**Data management, calculation, and statistics**

Acute reduction of lipoproteins after each treatment was calculated from systemic lipoprotein concentrations before and after each apheresis session (LDL-C_pre_ and LDLC_post_, respectively) using the following equation:

Eq. 1. % acute reduction of LDL-C = 100(LDL-C_pre_- LDLC_post_)/LDL-C_pre_.

Long-term reductions were calculated based on lipoprotein concentrations at baseline (LDL-Cbl), prior to the first treatment session, and the mean inter-apheresis level (LDL-Cmia) averaged from the last 3 sessions, as previously described. 

Eq. 2. % long-term reduction of LDL-C = 100(LDL-C_bl_ – LDL-C_mia_)/LDLC_bl_, 

Eq. 3. LDL-C_mia_ = 1/4(_LDL-Cpost(n-2_) + LDL-C_pre(n-1)_ + LDLC_post(n-1)_ + LDL-C_pre(n)_), 

where subscripts (n-2), (n-1), and (n) refer to the antepenultimate, penultimate, and final session, respectively. These formulas were used for all hematological and biochemical parameters (4). 

Statistical analysis was performed using SPSS v.15.0 (Chicago, IL, USA). The unit of analysis used here is the apheresis session, not the individual patient. The descriptives provided are averaged over all the three patients. Results are expressed as mean ± SD. Comparison of differences between pre- and post-treatment values was performed using the paired t test. All the variables were normally distributed which were determined by Kolmogorov- Smirnov test. A p-value of less than 0.05 was considered to be statisticallly significant. 

The local ethics committee approved the study.

## RESULTS

Patient 1 started LDL apheresis treatment 2 years beforethis manuscript was written. In total, the patient received50 sessions of LDL apheresis by cascade infiltration.Mean LDL-C levels before and after treatment were420 ± 83 dL^–1^ and 142 ± 41 mg/dL, respectively. Acuteand long-term mean reductions in LDL-C were statisticallysignificant (p= 0.010, p= 0.023). Acute mean reductionsin the other parameters were as follows: total cholesterol:58.2%; HDL-C: 42.3%; triglycerides: 57.1%; fibrinogen:55.4%; IgG: 31.4%; IgM: 18.4%; IgA: 14.2%. The xanthomasdecreased in size after 1 year, but did not completelydisappear.

Patient 2 underwent 35 sessions of LDL apheresis.Mean LDL-C levels before and after treatment were 353 ±63 dL^–1^ and 136 ± 34 mg/dL, respectively. Acute and longtermmean reductions in LDL-C were statistically significant(p= 0.011, p= 0.034). Acute mean reductions in theother parameters were as follows: total cholesterol: 61.3%;HDL-C: 40.4%: triglycerides: 60.9%; fibrinogen: 60.3%;28. IgG: 6%; IgM: 16.1%; IgA: 11.8%. Regular sessions ofLDL apheresis led to resolution of the xanthomas. 

Patient 3 also underwent 35 sessions of LDL apheresis.Mean LDL-C levels before and after treatment were 479 ±64 dL^–1^ and 158 ± 26 mg/dL, respectively. Acute and longtermmean reductions in LDL-C were statistically significant(p= 0.013, p= 0,032). Acute mean reductions in theother parameters were as follows: total cholesterol: 54.2%;HDL-C: 39.5%; triglycerides: 59.2%; fibrinogen: 63.4%;IgG: 31.4%; IgM: 17.1%; IgA: 10.9%. The majority of thexanthomas disappeared after 20 sessions.

The treatments were well tolerated by all the patients.The most frequently occurring technical problems wererelated to bloodlines; puncture difficulties (1.7%) during2 sessions and insufficient blood flow (13.5%) during 16sessions. The main clinical adverse effects were hypotensionduring 3 sessions (2.5%), chills (1.7%) during 2 sessions,and nausea and vomiting during 3 sessions (2.5%).

**Figure 3:**
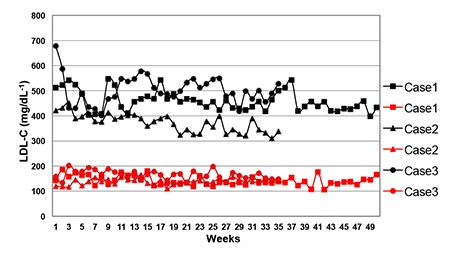
Pre-and post-treatment LDL-C levels

Mean session duration was 200 ± 30 min and meanblood volume treated ranged from 1,700 to 3,500 mLsession^–1^, depending on the body weight of the patient.Blood flow rate was 35-50 mL min^–1^. In all patients thepre-treatment mean LDL-C value was 418 ± 62 mg/dL,the post-treatment mean LDL-C value was 145 ± 43 (p=0.011), and the acute mean reduction in the LDL-C levelwas 70.8%. At the end of the treatment sessions LDL-C,HDL-C, total cholesterol, and triglyceride levels returnedto normal ranges, and the improvement in lipid profileswas significant (Table 2). Serum protein, albumin, andfibrinogen values decreased significantly after the eachtreatment session; however, this reduction in the serumprotein profile was not clinically important (Table 2). Areduction was observed in immunoglobulin levels, butdid not reach abnormal levels (Table 2). The hematologicalparameters did not change after treatment, except forplatelets, but the platelet counts remained within the normalrange (Table 2). Pre- and post-treatment LDL-C levelsare shown in Figure 3. 

## DISCUSSION

Elevated LDL-C causes angina pectoris, MI, and suddendeath during the second decade of life in homozygous FHpatients. Several studies have shown that long-term LDLapheresisfor homozygous FH patients prevents coronaryheart disease [1,14]. De Gennes first reported the effectivenessof plasma exchange in successfully reducing plasmacholesterol levels in 1967; it was well tolerated, but repetitionof the procedure was emotionally difficult for thepatients (15). In 1976 Lupien performed plasmapheresisto selectively remove LDL-C; subsequently, LDL apheresisreplaced plasma-exchange therapy [16]. The newprocedure was feasible, well tolerated, and had a greatereffect on clinical status. Recently, several new apheresistechniques were developed, including dextran sulphatecelluloseadsorption (DSA), the heparin extracorporealLDL precipitation system (HELLP), direct adsorption oflipoprotein using hemoperfusion (DALI), immunoadsorption,and cascade filtration [17,18,19,20]. Researchers reportedthat these technically different procedures were similarlyeffective in lowering serum LDL-C [21,22,23]. 

The American Society for Apheresis (ASFA) recommendscascade filtration plasmapheresis as a standard andacceptable primary treatment modality for FH (category1) [24]. With the cascade filtration system atherogenic lipoproteinsare eliminated from plasma, according to theirmolecular size. The cascade filter pore diameter is >15-30nm, which ensures that larger plasma proteins (lipoproteins,albumin, fibrinogen, etc.) are caught in the filter, allowingsmaller molecules to pass through [25]. In the presentstudy LDL-C levels significantly decreased; mean acutereduction in LDL-C was 70.8%, and reductions in other lipoproteinswere as follows: total cholesterol: 57.9%; HDLC:40.7%; triglycerides: 59.2%. The Coker [12] study reporteda reduction of 63% in mean plasma LDL-C usingthe cascade filtration technique in 10 patients. Zwiener[6] obtained a 71% reduction in mean plasma LDL-C usingthe DSA technique in 2 patients. In the Bosh study[4] acute mean reductions in lipoproteins achieved by theDALI system were as follows: LDL-C: 69%; triglycerides:27%; HDL-C: 11%; total cholesterol: 52%. Another studyperformed using the cascade filtration system reported thefollowing acute mean reductions: LDL: 61.6%; total cholesterol:59.5%; HDL-C: 31.1%; triglycerides: 48.1% [26]. 

Studies have shown that cascade filtration can causeundesirable reductions in other large-molecule proteins,such as immunoglobulins, serum total protein, albumin,and fibrinogen, but these levels remained within normalranges and were clinically unimportant. In the presentstudy the mean decrease in IgG was 30.9% and was withinthe normal range for the patients’ age group. Mean decreasein IgG ranged from 27% to 32% in other studies [25,27].Although acute mean reductions of 59.7% in fibrinogen,21.5% in serum protein, 14.7% in serum albumin, and14.2% in platelets were noted, we did not observe any infection,hemorrhagia, or edema in our patients. In a studyperformed using the DALI system acute mean reductionsof 15% in fibrinogen, 6% in platelets, and 8% in serumalbumin were reported [4]. Another study performed usingthe cascade filtration system reported mean reductionsof 49.9% in fibrinogen, 19.6% in albumin, and 26.9% inserum total protein [26]. 

Undesirable side effects rarely occur in patients treatedwith the cascade filtration method. In the REMUKASTstudy adverse reactions were reported in 34 of 1,702 treatments(2%), including hypotension (41%), nausea (18%),and edema (17%) [28]. Another study reported the followingclinical side effects: hypotension (0.2%), chills (0.1%),and nausea and vomiting (0.2%) [12]. We observed similarside effects in the present study (hypotension, chills, nausea and vomiting, etc.) and their frequency was nothigher than expected. 

In conclusion, the cascade filtration technique is a very effective treatment for removing undesirable lipoproteins, such as LDL-C, total cholesterol, and triglycerides, from plasma; it is safe, and is associated with minor adverse effects and negligible technical problems. 

**Conflict of Interest Statement **

The authors of this paper have no conflicts of interest, including specific financial interests, relationships, and/ or affiliations relevant to the subject matter or materials included.

## Figures and Tables

**Table 1:**
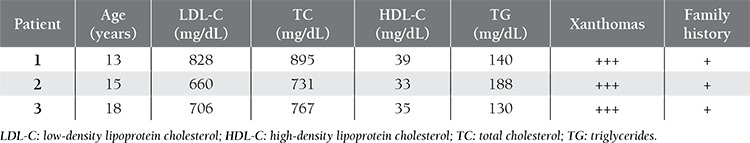
Family history, and laboratory and clinical features of the patients before LDL apheresis.

**Table 2:**
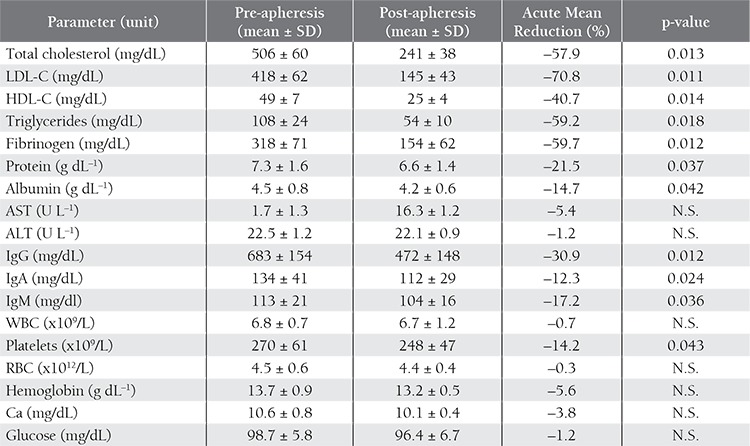
The effects of 120 cascade filtration sessions on hematological, lipid, and biochemical parameters in 3 patients.

**Figure 1a:**
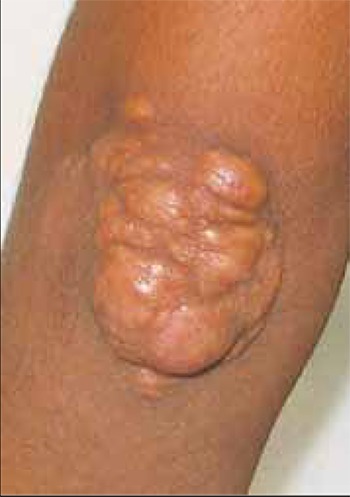
Pre-apheresis xanthomas on the right elbow of the first patient

**Figure 1b:**
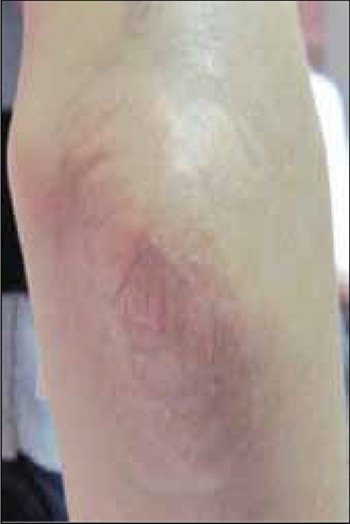
Post-apheresis xanthomas on the right elbow of the first patient.
